# PD-1/PD-L1 signaling in JIA synovial regulatory T cell function

**DOI:** 10.1186/1546-0096-9-S1-P279

**Published:** 2011-09-14

**Authors:** Sytze de Roock, Genoveva Keustermans, Chantal L Duurland, Ellen JM Wehrens, Femke van Wijk, Berent J Prakken

**Affiliations:** 1 Center for Molecular and Cellular Intervention CMCI, Pediatric Immunology, Wilhelmina Children’s Hospital Utrecht, The Netherlands

## 

Regulatory T cells (Treg) are key players in the prevention of aberrant immune responses and can be a future therapeutic target in auto-immunity. Tregs can be induced in the periphery by cytokines like TGFβ, or in the presence of inhibitory signals provided by antigen presenting cells. The murine programmed death (PD)-1 molecule is known to induce FOXP3 expression by influencing the downstream signaling of the T cell receptor and CD28 via inhibition of the PI3K/PKB pathway.

We investigated the role of PD-1/PD-L1 interaction for the induction and functioning of Juvenile Idiopathic Arthritis (JIA) patient synovial Treg. Expression profiles of surface molecules and FOXP3 were monitored by FACS analysis. Cells were in vitro activated by beads coated with anti-CD3/anti-CD28 and PD-L1 or isotype.

High numbers of PD-1+ T cells and PD-L1+ macrophages were found in the synovium of JIA patients. *In vitro* incubation of peripheral blood cells from healthy donors with synovial fluid from JIA patients induced upregulation of these molecules. Activation of CD4 T cells from healthy individuals by beads with PD-L1 resulted in reduced proliferation and increased induction of FOXP3+ cells, accompanied by a reduced PKB phosphorylation as compared to isotype. Culture of synovial T cells with PD-L1 coated beads did not influence the highly activated status of PKB, but did reduce the proliferation of these cells.

These data suggest an important role for PD-1 and its ligand in human Treg biology and immune homeostasis in chronically inflamed tissue which will be further investigated in future experiments.

